# Immunogenicity and safety of a trivalent inactivated influenza vaccine produced in Shenzhen, China versus a comparator influenza vaccine: a phase IV randomized study

**DOI:** 10.1080/21645515.2019.1581541

**Published:** 2019-04-02

**Authors:** Yuemei Hu, Kai Chu, Nathalie Lavis, Xiaoling Li, Bill Liang, Shuzhen Liu, Ming Shao, Jean-Denis Shu, Cynthia Tabar, Sandrine Samson

**Affiliations:** a Vaccine Clinical Evaluation Department, Jiangsu Center for Disease Prevention and Control, Nanjing, China; b Medical Operations, Sanofi Pasteur, Lyon, France; c Medical Operations, Sanofi Pasteur, Beijing, China; d Medical Department, Sanofi Pasteur, Beijing, China; e Division of Respiratory Virus Vaccines, National Institutes for Food and Drug Control, Beijing, China; f Medical Department, Sanofi Pasteur, Lyon, France; g Clinical Operations, Sanofi Pasteur, Lyon, France; h Global Medical, Sanofi Pasteur, Swiftwater, PA, USA

**Keywords:** Trivalent influenza vaccines, China, immunogenicity, safety, reactogenicity, influenza, vaccination

## Abstract

Seasonal influenza causes substantial morbidity and mortality in China, which largely results from limited vaccine accessibility and poor vaccination coverage. Since 2013, Sanofi Pasteur’s facilities in Shenzhen, China have produced a trivalent inactivated influenza vaccine (Shz-IIV3) for each influenza season according to Chinese pharmacopeia requirements. However, the immunogenicity of Shz-IIV3 has not been compared to existing Chinese trivalent inactivated influenza vaccines (IIV3s). Here, we describe the results of a phase IV, observer-blind, randomized study to evaluate whether the immunogenicity of Shz-IIV3 was non-inferior to a comparator IIV3 (Hualan Biological Engineering Inc) also manufactured and licensed in China. Healthy adults aged 18−59 years were randomly assigned in a 1:1 ratio to receive a single 0.5-mL intramuscular injection of the 2017–2018 Northern Hemisphere formulation of Shz-IIV3 (n = 800) or the comparator IIV3 (n = 799). Between baseline and day 28 after vaccination, hemagglutination inhibition titers for the three vaccine strains increased by at least 4-fold and were of similar magnitude in Shz-IIV3 and comparator IIV3 recipients. The rate of seroconversion or significant increase in titers was 62% to 92% in Shz-IIV3 recipients, and 63% to 91% in comparator IIV3 recipients. Post-vaccination hemagglutination inhibition titers and seroconversion rates for Shz-IIV3 were statistically non-inferior to the comparator IIV3 for all three influenza vaccine strains. Rates of solicited and unsolicited vaccine-related adverse events were similar between the two vaccine groups. These results demonstrated that Shz-IIV3 was as immunogenic and safe in adults as a comparator Chinese IIV3, and support the continued use of Shz-IIV3 in China.

Seasonal influenza is a serious public health concern that causes substantial mortality and morbidity, particularly among older adults, children aged less than 5 years, and persons with chronic medical conditions.^1^ In China, seasonal influenza causes an estimated annual mortality rate of 11.1−12.4 per 100,000^2,3^ and hospitalization rates for severe acute respiratory infection of 115−142 individuals per 100,000.^4^

Vaccination is the most effective way to prevent influenza.^5^ Consistent with World Health Organization recommendations,^6^ seasonal vaccination is recommended in China for those at greatest risk from influenza complications.^7^ However, despite these recommendations, less than 10% of at-risk individuals are vaccinated in the country in each season.^8^ The low vaccine coverage is likely due to the exclusion of influenza vaccines from the national immunization program, meaning that individuals must pay for the vaccine voluntarily.^7,9^ Consequently, new policies are being explored in China to improve vaccine accessibility among at-risk populations, such as free vaccination for older persons.^9^

Since 2013, a split virion trivalent inactivated influenza vaccine (IIV3) has been produced by the Sanofi Pasteur facilities based at Shenzhen, China (Shz-IIV3) according to Chinese pharmacopeia requirements.^10^ Shz-IIV3 is well tolerated and highly immunogenic in Chinese individuals aged ≥ 6 months,^10^ but its immunogenicity and safety compared to existing IIV3s in China have not been assessed. Here we report the results of an observer-blind randomized study to evaluate whether the immunogenicity of Shz-IIV3 was non-inferior to a comparator IIV3 also manufactured in China (Hualan trivalent influenza vaccine [split virion, inactivated]; Hualan Biological Engineering Inc, China) in healthy adults aged 18–59 years.

The study enrolled 1600 adults between November 3 and December 23, 2017, who were randomized 1:1 to receive either Shz-IIV3 (n = 800) or the comparator IIV3 (n = 799). Each vaccine contained 15 µg hemagglutinin per strain of A/Michigan/45/2015 (H1N1)pdm09, A/Hong Kong/4801/2014 (H3N2), and B/Brisbane/60/2008 (the 2017–2018 Northern Hemisphere formulation), without any additional component or adjuvant. One subject withdrew consent before being randomized or vaccinated. Most subjects in each group were female (62.3% of Shz-IIV3 recipients and 63.3% of comparator IIV3 recipients) and the median ages were 46.2 years (range: 19.8−62.5 years) in the Shz-IIV3 group and 46.3 years (range: 18.0−59.9 years) in the comparator IIV3 group. None of the subjects had received influenza vaccination in the previous (2016−2017) season.

Eight vaccinated subjects (three Shz-IIV3 recipients and five comparator IIV3 recipients) were excluded from the immunogenicity analyses due to non-compliance with the study’s inclusion or exclusion criteria, or missing blood samples. At baseline, the proportions of seronegative subjects were similar between the two groups for all vaccine strains (Table 1). Twenty-eight days after vaccination, all subjects had detectable hemagglutination inhibition (HAI) antibody titers against the three vaccine strains, except for seven subjects (0.9%) vaccinated with the comparator IIV3 that did not have detectable titers against A/H1N1. Shz-IIV3 increased HAI geometric mean titers (GMTs) for all vaccine strains by 4-fold to 32-fold over baseline GMTs, and at least 99% of Shz-IIV3 recipients had titers ≥1:40. Similarly, the comparator IIV3 increased HAI GMTs by 4-fold to 35-fold, and at least 96% of recipients had titers ≥1:40. The rate of seroconversion or significant increase in titer was 62% to 92% in Shz-IIV3 vaccine recipients, and 63% to 91% in comparator IIV3 recipients. These results are in line with an earlier study that showed the 2014–2015 Northern Hemisphere formulation of Shz-IIV3 was highly immunogenic in Chinese individuals ≥6 months of age.^10^

**Table 1.** Humoral immunogenicity.

		A/H1N1		A/H3N2		B/Brisbane	
Measure	Day	Shz-IIV3	Comparator IIV3	Shz-IIV3	Comparator IIV3	Shz-IIV3	Comparator IIV3
N	-	797	794	797	794	797	794
HAI GMT (95% CI)	0	20.9 (19.3; 22.6)	21.7 (20.1; 23.5)	46.2 (43.7; 48.9)	47.5 (45.0; 50.2)	43.3 (41.1; 45.6)	43.0 (40.8; 45.4)
	28	669 (614; 729)	760 (685; 843)	213 (198; 229)	236 (219; 253)	392 (364; 422)	364 (338; 392)
HAI titer ≥1:40, % (95% CI)	0	37.5 (34.1; 41.0)	39.5 (36.1; 43.0)	75.2 (72.0; 78.1)	76.6 (73.5; 79.5)	71.6 (68.4; 74.8)	71.8 (68.5; 74.9)
	28	98.9 (97.9; 99.5)	95.8 (94.2; 97.1)	99.1 (98.2; 99.6)	99.5 (98.7; 99.9)	100.0 (99.5; 100.0)	99.7 (99.1; 100.0)
GMTR (95% CI)	28/0	32.1 (28.6; 36.0)	35.0 (31.4; 39.1)	4.61 (4.26; 4.98)	4.95 (4.56; 5.38)	9.05 (8.37; 9.79)	8.46 (7.80; 9.17)
Seroconversion^a^, % (95% CI)	28/0	91.7 (89.6; 93.5)	90.9 (88.7; 92.8)	61.7 (58.3; 65.1)	62.8 (59.4; 66.2)	84.8 (82.1; 87.2)	(78.6; 84.1)

This was a phase IV, observer-blind, randomized study conducted at a single center in the Province of Jiangsu, China between November 2017 and May 2018 (WHO Universal Trial Number: U1111-1183-5912). The study was conducted at the request of the China Food and Drug Administration as part of a license renewal commitment. Healthy adults aged 18−59 years were randomly assigned in a 1:1 ratio to receive a single 0.5-mL intramuscular injection of Shz-IIV3 (Sanofi Pasteur; n = 800) or the comparator IIV3 (Hualan trivalent influenza vaccine [split virion, inactivated]; Hualan Biological Engineering Inc, China; n = 799). Each 0.5-mL dose contained 15 µg hemagglutinin per strain of A/Michigan/45/2015 (H1N1)pdm09, A/Hong Kong/4801/2014 (H3N2), and B/Brisbane/60/2008 (the 2017–2018 Northern Hemisphere formulation). The study ended on May 22, 2018 and was completed by all randomized subjects except for one Shz-IIV3 recipient who died more than 3 months after vaccination due to an unrelated serious adverse event. Further details of the study ethics, exclusion criteria, randomization and blinding methods, and calculation of sample size are provided in the **Supplemental Online Information**. Blood samples were taken at baseline (day 0) and 28 days after vaccination for immunogenicity assessment. Immunogenicity was measured in all subjects completing the study according to protocol as described previously^11^ and using the recommendations of the CHMP Note for Guidance on Harmonization of Requirements for Influenza Vaccines.^12^ 95% CIs were calculated using the exact binomial method (Clopper-Pearson method). Abbreviations: CI, confidence interval; GMT, geometric mean titer; GMTR, geometric mean of the individual ratios of the post-vaccination (day 21) HAI titer divided by the pre-vaccination (day 0) HAI titer; HAI, hemagglutination inhibition; IIV3, trivalent inactivated influenza vaccine; Shz-IIV3, trivalent inactivated influenza vaccine produced in Shenzhen, China.

^a^ Seroconversion was defined as a pre-vaccination (day 0) HAI titer <1:10 and a post-vaccination (day 28) HAI titer ≥1:40, or as a pre-vaccination HAI titer ≥1:10 and a ≥ 4-fold increase in HAI titer

Post-vaccination HAI GMTs and seroconversion rates for Shz-IIV3 were statistically non-inferior to those for the comparator IIV3, for all three vaccine strains (Table 2). Because non-inferiority was shown for all six analyses, Shz-IIV3 was considered to demonstrate overall non-inferiority versus the comparator vaccine.

**Table 2.** Non-inferiority analysis using post-vaccination (day 28) GMTs and seroconversion rates for Shz-IIV3 vs. the comparator IIV3.

Measure	Strain	Value (95% CI)^c^	Non-inferior
Ratio of day 28 HAI GMTs ^a^	A/H1N1	0.88 (0.77; 1.01)	Yes
	A/H3N2	0.90 (0.82; 1.00)	Yes
	B Victoria lineage	1.08 (0.97; 1.20)	Yes
Difference in seroconversion, % ^b^	A/H1N1	0.79 (−2.00; 3.58)	Yes
	A/H3N2	−1.11 (−5.86; 3.64)	Yes
	B Victoria lineage	3.33 (−0.35; 7.01)	Yes

For each vaccine strain, non-inferiority of the HAI antibody response induced by Shz-IIV3 versus the comparator IIV3 was determined from post-vaccination GMTs and seroconversion rates, according to the non-inferiority margin guideline of the European Medicine Agency Committee for Medicinal Products for Human Use.^13^ Overall non-inferiority was demonstrated if non-inferiority was concluded for all six individual analyses. Non-inferiority was assessed in all subjects completing the study according to protocol. Data were analyzed using SAS® version 9.4 (SAS Institute). Abbreviations: CI, confidence interval; GMT, geometric mean titer; HAI, hemagglutination inhibition.

^a^ For each strain, non-inferiority was concluded if the lower limit of the two-sided 95% CI of the ratio of day 28 GMTs between Shz-IIV3 and comparator IIV3 was >0.667

^b^ For each strain, non-inferiority was concluded if the lower limit of the two-sided 95% CI of the difference of seroconversion rates between Shz-IIV3 and comparator IIV3 was > −10%

^c^ Two-sided 95% CIs are calculated based on the Student t-distribution for non-inferiority analysis of GMTs, and based on the Wilson score method^14^ for non-inferiority analysis of seroconversion rate differences

Solicited injection site reactions were reported by similar proportions of subjects in the Shz-IIV3 and comparator IIV3 groups (31.4% vs. 33.0%), as were solicited systemic reactions (12.8% vs. 12.1%) (Table 3). Consistent with other studies of IIV3,^10,16^ the most frequently reported solicited reactions in each group were malaise, myalgia, and pain and erythema at the injection site (Supplemental Table 1). For both groups, most solicited reactions were grade 1 (mild) in intensity and most resolved within 3 days. Few subjects reported grade 3 (severe) solicited reactions (2.0% of Shz-IIV3 recipients and 1.5% of comparator IIV3 recipients; data not shown), which were mostly injection-site reactions that resolved spontaneously.

**Table 3.** Adverse events after vaccination.

	Shz-IIV3		Comparator IIV3	
Subjects experiencing at least one:	(N = 803)^a^		(N = 796)^a^	
	n	% (95% CI)	n	% (95% CI)
Immediate unsolicited AE	2	0.2 (0.0; 0.9)	0	0.0 (0.0; 0.5)
Vaccine-related	1	0.1 (0.0; 0.7)	0	0.0 (0.0; 0.5)
Solicited reaction	277	34.5 (31.2; 37.9)	290	36.4 (33.1; 39.9)
Solicited injection-site reaction	252	31.4 (28.2; 34.7)	263	33.0 (29.8; 36.4)
Solicited systemic reaction	103	12.8 (10.6; 15.3)	96	12.1 (9.9; 14.5)
Unsolicited AE	32	4.0 (2.7; 5.6)	30	3.8 (2.6; 5.3)
Non-serious	32	4.0 (2.7; 5.6)	29	3.6 (2.5; 5.2)
Non-serious vaccine-related	14	1.7 (1.0; 2.9)	9	1.1 (0.5; 2.1)
Injection-site non-serious vaccine-related	3	0.4 (0.1; 1.1)	0	0.0 (0.0; 0.5)
Systemic non-serious vaccine-related	11	1.4 (0.7; 2.4)	9	1.1 (0.5; 2.1)
AE leading to study discontinuation	0	0.0 (0.0; 0.5)	0	0.0 (0.0; 0.5)
SAE within 6 months	5	0.6 (0.2; 1.4)	6	0.8 (0.3; 1.6)
Vaccine-related	0	0.0 (0.0; 0.5)	0	0.0 (0.0; 0.5)
Death within 6 months^b^	1	0.1 (0.0; 0.7)	0	0.0 (0.0; 0.5)

Safety was analyzed in all subjects who received a study vaccine and was assessed according to the International Conference on Harmonisation E2A Guideline for Clinical Safety Data Management: Definitions and Standards for Expedited Reporting^15^ Subjects recorded information about solicited reactions in a diary card for up to 7 days after vaccination, and about unsolicited AEs up to 28 days after vaccination. Any SAEs were reported to investigators throughout a 6-month safety follow-up. Investigators assessed unsolicited AEs and SAEs as unrelated or possibly related to the vaccination. Immediate unsolicited AEs were defined as those occurring within 30 min following vaccination. AEs were coded with Medical Dictionary for Regulatory Activities (MedDRA) terminology (version 19.0). 95% CIs were calculated using the exact binomial method (Clopper-Pearson method). Abbreviations: AE, adverse event; CI, confidence interval; IIV3, trivalent inactivated influenza vaccine; SAE, serious adverse event; Shz-IIV3, trivalent inactivated influenza vaccine produced in Shenzhen, China.

^a^ Safety data were analyzed according to the vaccine received. Due to errors in vaccine allocation, 803 subjects received Shz-IIV3 (799 randomized to the Shz-IIV3 group and 4 randomized to the comparator group) and 796 subjects received the comparator IIV3 (1 randomized to the Shz-IIV3 group and 795 randomized to the comparator IIV3 group)

^b^ Not considered vaccine-related

The proportions of subjects reporting unsolicited vaccine-related adverse events (AEs) were also similar between the Shz-IIV3 and comparator IIV3 groups (1.7% [95% confidence interval: 1.0−2.9%] vs. 1.1% [0.5−2.1%]) (Table 3). These were mostly cases of nasopharyngitis, cough, and injection site pruritus (Supplemental Table 1). One subject vaccinated with Shz-IIV3 experienced an immediate unsolicited AE within 30 minutes after vaccination (dizziness), which was assessed by the investigator as related to the vaccine. The subject recovered spontaneously within the same day. Rare cases of dizziness have been reported for other influenza vaccines in China and elsewhere, and, as seen in the present study, these cases usually resolved spontaneously within hours of onset.^17-19^ No AEs led to discontinuation within 28 days after vaccination. None of the serious AEs reported during the study, or the death that occurred >3 months after vaccination, were considered vaccine-related.

These results showed that the immunogenicity and safety of a marketed influenza vaccine, Shz-IIV3, were similar to the comparator Chinese IIV3 in individuals aged 18−59 years. No new safety signals were seen for either vaccine and both were well tolerated. This was the largest study to evaluate Shz-IIV3 in China so far. Although the study did not include individuals aged <5 years and ≥60 years who are considered at greater risk,^1^ the vaccine was recently shown to be immunogenic and well tolerated in these age groups.^10^ Demonstration of the high immunogenicity and safety of Shz-IIV3 should help encourage its continued use in China.
