# Supplementary material for: Immunogenicity and safety of a trivalent inactivated influenza vaccine produced in Shenzhen, China versus a comparator influenza vaccine: a phase IV randomized study
Source: Hum Vaccin Immunother. 2019 Apr 2;15(5):1066–9. doi: 10.1080/21645515.2019.1581541 (PMC6605815; doi:10.1080/21645515.2019.1581541)
Supplement: Supplemental Material [file khvi-15-05-1581541-s001.docx]

# Supplementary Online Information for “Immunogenicity and safety of a trivalent inactivated influenza vaccine produced in Shenzhen, China versus a comparator influenza vaccine: a phase IV randomized study” by Hu et al.

## Ethics

The study was approved by the Ethics Committee of Jiangsu Provincial Center for Disease Prevention and Control and conducted in accordance with the Declaration of Helsinki, the International Conference on Harmonisation Guidelines for Good Clinical Practice, and local regulations and directives. All subjects provided their written informed consent.

## Exclusion criteria

Potential subjects were excluded if they had: received any vaccine in the 2 weeks preceding study vaccination or planned to receive any vaccine in the 2 weeks following study vaccination; been vaccinated against influenza within 6 months before inclusion; received immune globulins, blood, or blood-derived products in the preceding 3 months; known hypersensitivity or history of a life-threatening reaction to the study vaccines or their components; known or suspected congenital or acquired immunodeficiency; received immunosuppressive therapy within the preceding 6 months; received long-term systemic corticosteroid therapy (prednisone or equivalent for >2 consecutive weeks) within the past 3 months; received oral or injectable antibiotic therapy within 72 hours prior to the first blood draw; had thrombocytopenia, a bleeding disorder, or had received anticoagulants in the 3 weeks before the study; had a chronic illness that, in the opinion of the investigator, might interfere with the study assessments; or had moderate or severe acute illness or infection on the day of vaccination or febrile illness (axillary temperature ≥37.1°C). Women were excluded if they were pregnant, lactating, or of childbearing potential and not using contraception or abstinent.

## Randomization and blinding methods

Subjects were randomly assigned in a 1:1 ratio to receive a single dose (0.5 ml) of Shz-IIV3 or the comparator IIV3. Randomization was performed by the permuted block method using a computer-generated randomization code supplied by the Sponsor. Scratchable randomization lists were used to communicate to the vaccine administrator which vaccine was to be injected. Study participants and the investigator responsible for assessing safety within 30 minutes of vaccination were blinded to which vaccine was administered. To keep the blind, vaccine preparation and administration, and safety assessment were performed in different rooms by different individuals.

## Sample size

1600 subjects were to be enrolled to randomize 800 subjects in each vaccine group. This produced an overall power of >90% to demonstrate: (1) non-inferiority of geometric mean titers induced by Shz-IIV3 versus the comparator IIV3 with a non-inferiority margin of 1.5, assuming a standard deviation of log_10_-transformed titers of 0.7 for each A strain and 0.6 for the B strain, and a 10% drop-out rate; (2) non-inferiority of seroconversion rates from Shz-IIV3 versus the comparator IIV3 with a non-inferiority margin of 10%, proportions in the control group of 0.55 for each A strain and 0.50 for the B strain, a true difference in proportions of 0, and a 10% drop-out rate. Additionally, the Shz-IIV3 population size (n=800) provided a probability of approximately 95% for observing any adverse event with a true incidence of 0.37%.

# Supplementary tables

**Supplementary Table 1: Solicited reactions and vaccine-related unsolicited adverse events**

|  | **Shz-IIV3** | |  | **Comparator IIV3** | |  |
| --- | --- | --- | --- | --- | --- | --- |
|  | **(N=803)^a^** | |  | **(N=796)^a^** | |  |
| **Subjects experiencing at least one:** | **n** | **% (95% CI)** |  | **n** | **% (95% CI)** |  |
| Solicited injection-site reactions | 252 | 31.4 (28.2; 34.7) |  | 263 | 33.0 (29.8; 36.4) |  |
| Injection-site pain | 229 | 28.5 (25.4; 31.8) |  | 231 | 29.0 (25.9; 32.3) |  |
| Injection site erythema | 65 | 8.1 (6.3; 10.2) |  | 77 | 9.7 (7.7; 11.9) |  |
| Injection site swelling | 57 | 7.1 (5.4; 9.1) |  | 60 | 7.5 (5.8; 9.6) |  |
| Injection site induration | 58 | 7.2 (5.5; 9.2) |  | 54 | 6.8 (5.1; 8.8) |  |
| Injection site ecchymosis | 12 | 1.5 (0.8; 2.6) |  | 16 | 2.0 (1.2; 3.2) |  |
|  |  |  |  |  |  |  |
| Solicited systemic reactions | 103 | 12.8 (10.6; 15.3) |  | 96 | 12.1 (9.9; 14.5) |  |
| Fever | 20^b^ | 2.5 (1.5; 3.8) |  | 20^c^ | 2.5 (1.5; 3.9) |  |
| Headache | 29 | 3.6 (2.4; 5.1) |  | 31 | 3.9 (2.7; 5.5) |  |
| Malaise | 55 | 6.8 (5.2; 8.8) |  | 55 | 6.9 (5.2; 8.9) |  |
| Myalgia | 47 | 5.9 (4.3; 7.7) |  | 40 | 5.0 (3.6; 6.8) |  |
| Shivering | 8 | 1.0 (0.4; 2.0) |  | 12 | 1.5 (0.8; 2.6) |  |
|  |  |  |  |  |  |  |
| Vaccine-related unsolicited AEs | 14 | 1.7 (1.0; 2.9) |  | 9 | 1.1 (0.5; 2.1) |  |
| Injection-site pruritus | 3 | 0.4 (0.1; 1.1) |  | 0 | 0.0 (0.0; 0.5) |  |
| Nasopharyngitis | 7 | 0.9 (0.4; 1.8) |  | 4 | 0.5 (0.1; 1.3) |  |
| Tonsillitis | 1 | 0.1 (0.0; 0.7) |  | 0 | 0.0 (0.0; 0.5) |  |
| Upper respiratory tract infection | 0 | 0.0 (0.0; 0.5) |  | 1 | 0.1 (0.0; 0.7) |  |
| Dizziness | 1 | 0.1 (0.0; 0.7) |  | 0 | 0.0 (0.0; 0.5) |  |
| Cough | 1 | 0.1 (0.0; 0.7) |  | 3 | 0.4 (0.1; 1.1) |  |
| Oropharyngeal pain | 0 | 0.0 (0.0; 0.5) |  | 1 | 0.1 (0.0; 0.7) |  |
| Rhinorrhea | 1 | 0.1 (0.0; 0.7) |  | 0 | 0.0 (0.0; 0.5) |  |

Abbreviations: AE, adverse event; CI, confidence interval; IIV3, trivalent inactivated influenza vaccine; SAE, serious adverse event; Shz-IIV3, trivalent inactivated influenza vaccine produced in Shenzhen, China

^a^ Safety data were analyzed according to the vaccine received. Due to errors in vaccine allocation, 803 subjects received Shz-IIV3 (799 randomized to the Shz-IIV3 group and 4 randomized to the comparator IIV3 group) and 796 subjects received the comparator IIV3 (1 randomized to the Shz-IIV3 group and 795 randomized to the comparator IIV3 group)

^b^ N=800

^c^ N=794
